# Development and Evaluation of a Natural Language Processing Annotation Tool to Facilitate Phenotyping of Cognitive Status in Electronic Health Records: Diagnostic Study

**DOI:** 10.2196/40384

**Published:** 2022-08-30

**Authors:** Ayush Noori, Colin Magdamo, Xiao Liu, Tanish Tyagi, Zhaozhi Li, Akhil Kondepudi, Haitham Alabsi, Emily Rudmann, Douglas Wilcox, Laura Brenner, Gregory K Robbins, Lidia Moura, Sahar Zafar, Nicole M Benson, John Hsu, John R Dickson, Alberto Serrano-Pozo, Bradley T Hyman, Deborah Blacker, M Brandon Westover, Shibani S Mukerji, Sudeshna Das

**Affiliations:** 1 Department of Neurology Massachusetts General Hospital Boston, MA United States; 2 Harvard Medical School Boston, MA United States; 3 Vaccine and Immunotherapy Center Division of Infectious Disease Boston, MA United States; 4 Division of Pulmonary and Critical Care Medicine Massachusetts General Hospital Boston, MA United States; 5 Division of Infectious Diseases Massachusetts General Hospital Boston, MA United States; 6 Mongan Institute Massachusetts General Hospital Boston, MA United States; 7 McLean Hospital Belmont, MA United States; 8 Department of Psychiatry Massachusetts General Hospital Boston, MA United States

**Keywords:** chart review, cognition, cognitive status, dementia, diagnostic, electronic health record, health care, natural language processing, research cohort

## Abstract

**Background:**

Electronic health records (EHRs) with large sample sizes and rich information offer great potential for dementia research, but current methods of phenotyping cognitive status are not scalable.

**Objective:**

The aim of this study was to evaluate whether natural language processing (NLP)–powered semiautomated annotation can improve the speed and interrater reliability of chart reviews for phenotyping cognitive status.

**Methods:**

In this diagnostic study, we developed and evaluated a semiautomated NLP-powered annotation tool (NAT) to facilitate phenotyping of cognitive status. Clinical experts adjudicated the cognitive status of 627 patients at Mass General Brigham (MGB) health care, using NAT or traditional chart reviews. Patient charts contained EHR data from two data sets: (1) records from January 1, 2017, to December 31, 2018, for 100 Medicare beneficiaries from the MGB Accountable Care Organization and (2) records from 2 years prior to COVID-19 diagnosis to the date of COVID-19 diagnosis for 527 MGB patients. All EHR data from the relevant period were extracted; diagnosis codes, medications, and laboratory test values were processed and summarized; clinical notes were processed through an NLP pipeline; and a web tool was developed to present an integrated view of all data. Cognitive status was rated as cognitively normal, cognitively impaired, or undetermined. Assessment time and interrater agreement of NAT compared to manual chart reviews for cognitive status phenotyping was evaluated.

**Results:**

NAT adjudication provided higher interrater agreement (Cohen κ=0.89 vs κ=0.80) and significant speed up (time difference mean 1.4, SD 1.3 minutes; *P*<.001; ratio median 2.2, min-max 0.4-20) over manual chart reviews. There was moderate agreement with manual chart reviews (Cohen κ=0.67). In the cases that exhibited disagreement with manual chart reviews, NAT adjudication was able to produce assessments that had broader clinical consensus due to its integrated view of highlighted relevant information and semiautomated NLP features.

**Conclusions:**

NAT adjudication improves the speed and interrater reliability for phenotyping cognitive status compared to manual chart reviews. This study underscores the potential of an NLP-based clinically adjudicated method to build large-scale dementia research cohorts from EHRs.

## Introduction

In recent years, electronic health records (EHRs) have become increasingly common in US health care facilities; they provide a wealth of information on patient demographics, medical history, clinical data, and health system interactions. EHRs offer an unprecedented opportunity to improve clinical care and examine a broad variety of scientific, health care utilization, and heath policy questions [1-3]. An important first step in conducting EHR research is accurately identifying patients with a certain health condition, event, or disease, which is known as phenotyping [1,4]. The identified patient sample is subsequently leveraged for a wide range of purposes, such as providing clinical decision support for health care delivery [5], conducting epidemiological research [4,6], and for the practice of precision medicine [7].

Phenotyping cognitive status (ie, distinguishing between normal cognition and any stage of cognitive impairment) in EHR is a major challenge since dementia is underrecognized, underdiagnosed, and underreported in claims data [8-12], leading to inaccurate identification of dementia cases in many studies based on claims or EHR data [13-15]. Informative missingness, errors, and biases in EHR may further exacerbate the challenges of defining dementia outcomes [16]. Yet another challenge of phenotyping arises from complex, subjective, loosely-defined diagnostic criteria as well as the format—that is, structured (eg, diagnosis codes and medications) versus unstructured (eg, clinical notes and images)—in which the information is stored [4]. Previous studies have demonstrated that information on cognitive status is often found only in free text [17-19]. Clinicians may chart symptoms of cognitive problems in clinical notes but may not make a formal diagnosis, refer to a specialist, or prescribe medication for multiple reasons including clinical role, lack of time or expertise, patient resistance, or limited treatment options [20-22]. Thus, accurately phenotyping cognitive status requires the combined use of both structured data, such as diagnosis codes, medications, and laboratory test results, as well as unstructured clinical notes.

Several algorithms have been developed for phenotyping cognitive status; some studies used structured data, such as diagnosis codes, missed appointments, or health care utilization patterns [15,23], whereas others have applied natural language processing (NLP) to unstructured notes [18,19,24]. None of these prior efforts combined both structured and unstructured input modalities, and manual annotation by clinical experts is limited by the lack of available tools to facilitate efficient chart review [25]. Thus, we hypothesized that the best approach for phenotyping cognitive status is a semiautomated one in which automated NLP is applied to clinical notes and presented in an integrated view to the clinical expert for final manual adjudication of cognitive status.

We developed NAT, a semiautomated NLP-powered annotation tool, to facilitate adjudication of cognitive status. The tool extracts and processes data from EHRs and then ranks clinical notes based on a deep learning NLP algorithm (Macro *F*_1_=0.92) that classifies whether a note indicates normal cognition, cognitive impairment, or has no pertinent information [26]. It highlights key information and presents a summarized view to the annotator. We evaluated NAT in two EHR data sets: (1) Medicare beneficiaries from the Mass General Brigham (MGB) Accountable Care Organization (ACO) who were labeled in another study using manual chart reviews [15] and (2) MGB patients with laboratory confirmed SARS-CoV-2 (a case-control study to investigate the effects of COVID-19 on people with and without HIV was used as an exemplar of a research cohort that requires labeling of cognitive status). We evaluated interrater agreement in the first data set and compared it to interrater agreement in Epic—the EHR system used at MGB since 2015. The second data set was used to compare timings of manual to NAT adjudication, as the timing of manual adjudication was not available in the first data set.

By addressing the gaps in current chart review methods and leveraging existing NLP methods, we demonstrate that NAT increases both the efficiency and the interrater reliability of phenotyping cognitive status in EHR (relative to manual chart reviews) to build future research cohorts.

## Methods

### Clinical Settings and Data Sources

This diagnostic study was conducted at MGB—formerly Partners Healthcare—a private nonprofit organization comprising two major academic hospitals, community hospitals, and community health centers in the Boston metropolitan area. Data were sourced from the MGB Enterprise Data Warehouse that stores data from Epic. We evaluated NAT adjudication for phenotyping cognitive status on two distinct data sets. The first one included EHR data from January 1, 2017, to December 31, 2018, of 100 patients randomly selected from a larger data set that was expert-annotated via manual Epic chart reviews and reported elsewhere [15]. Specifically, this manually expert-annotated data set contained 1002 Medicare beneficiaries from the MGB ACO who were classified into (1) normal cognition, (2) borderline of normal cognition and mild cognitive impairment (MCI), (3) MCI, (4) borderline of MCI and dementia, or (5) dementia [15]. The experts graded their confidence in the adjudication as low, medium, moderate, or high. The 100 patients were randomly sampled from these 5 classes with 20 from each class, ensuring that each class had a similar distribution of confidence scores. The second data set included 527 MGB patients with a laboratory confirmed SARS-CoV-2 infection based on polymerase chain reaction testing between March 1 and December 31, 2020. The data set was created for a case-control study to investigate the effects of COVID-19 on people with and without HIV; EHR data up to 2 years prior to and any time after the index positive polymerase chain reaction test were used to investigate the performance of NAT adjudication.

### Ethics Approval

This study was approved by the MGB Institutional Review Board (2015P001915).

### Definition of Cognitive Impairment

In this study, to phenotype cognitive status, patients were annotated with three labels: (1) cognitively normal (CN), (2) cognitively impaired (CI), and (3) undetermined. Patients were labeled as CI if there was any documented suspicion or concern of memory or cognitive decline, whether based on symptoms, observations, or objective testing. This ranged from any dementia-related International Classification of Diseases (ICD) codes or medicines in the patients’ charts to cognitive concerns—relayed by patients, family members or friends, or providers in the notes and phone logs—as these concerns often reflect an underlying change in cognition even if a cognitive evaluation is normal (in which case they prompt a diagnosis of subjective cognitive decline [27]). Conversely, to be annotated as CN, at least implicit evidence of no cognitive concerns was required (eg, the patient continued to work, clearly managed their own care or hobbies, and followed complicated instructions, or they had annual wellness or specialist notes with multisystem assessment and no mention of a cognitive concern). The strongest evidence for a CN annotation was a cognition test performed with an explicit note of intact cognition. If there was conflicting evidence of both cognitive impairment and evidence of no cognitive impairment in a patient’s chart, the latest evidence or specialist notes (if any were available) informed the adjudication. Finally, patients were marked as “undetermined” if the EHR did not have sufficient information.

### Data Preparation

Data query, preparation, and preprocessing steps are described in Multimedia Appendix 1. For each patient, the following EHR data from the relevant time period were extracted from the Enterprise Data Warehouse: (1) patient demographic information, including name, medical record number, birth date, sex, ethnic group, marital status, and educational level; (2) all clinical notes, including reason for visit, history, note text, encounter type, and MGB provider (including provider department, specialty, and qualifications); (3) current primary care provider; (4) patient care coordination note; (5) medication history and current medications; (6) magnetic resonance imaging and computerized tomography orders; (7) laboratory orders and results; (8) problem list, including ICD diagnoses and diagnosis codes; and (9) visit cancellations.

Several features were engineered from the EHR to facilitate assessment of cognitive status. Dementia-related medications and ICD codes (medications: galantamine, donepezil, rivastigmine, and memantine; ICD-9 codes: 290.X, 294.X, 331.X, and 780.93; ICD-10 codes: G30.X and G31.X) and laboratory tests (eg, vitamin B12, folate, and thyroid-stimulating hormone) related to assessment of cognitive status were identified and highlighted. The numbers of cancellations, no-shows, and refill requests, relative to the total number of encounters, were computed.

Finally, NLP was applied to the clinical notes. We curated two lists of regular expressions or keywords related to the presence or absence of both (1) cognitive impairment and (2) the functional impairment of activities of daily living (ADLs) or independent ADLs, respectively (Multimedia Appendices 2 and 3). We identified regular expression matches and highlighted these within the text of the notes with different colors for each category (eg, cognition vs ADLs) to facilitate their identification by the clinician. We applied a previously developed NLP model [26] to generate classification probabilities of the following classes for each note: CI, no CI, or neither. The notes were ranked based on these classification probabilities, and notes that the model predicted as indicative of CI were displayed at the top.

### Development of an Annotation Tool

We designed and developed a web-based chart review and annotation tool, using the Python-based open-source Django web development framework with a SQLite database. We established data models for patient-level demographic and clinical data, encounter-level clinician notes, user account creation and authentication, and patient assignment to individual or multiple annotators (Multimedia Appendix 4). We created several user interfaces (ie, pages) to present the various data modalities in an integrated fashion for annotation.

### Statistical Analysis

We evaluated NAT adjudication using three metrics: agreement with manual Epic chart reviews, assessment time, and interrater agreement. We evaluated agreement between manual Epic chart reviews and NAT adjudication as well as interrater agreement for NAT adjudication using Cohen κ, whereas assessment time in minutes was compared using a paired samples Wilcoxon test (also known as the Wilcoxon signed-rank test). There were no missing data for these variables. All analyses were conducted using the R statistical software (version 4.1.2; R Core Team).

## Results

### Patient Characteristics

The patient characteristics of the two data sets are shown in Table 1. The ACO data set comprised 100 patients (63/100, 63.0% were women; mean age 78.8, SD 7.4 years; 7/100, 7% racial or ethnic minorities, 1 missing; 51/100, 51.0% with a college degree or more, 3 missing; and 50/100, 50.0% were married). The COVID-19 data set comprised 527 patients (226/527, 42.9% women; mean age 52.6, SD 15.0 years; 318/527, 60.35% racial or ethnic minorities, 21 missing; 160/527, 30.4% college education or more, 62 missing; and 195/527, 37.0% married, 16 missing).

**Table 1:** Characteristics of Accountable Care Organization (ACO) and COVID-19 data sets used for NLP^a^ annotation tool (NAT) evaluation.

Characteristics			Patients (N=627)		
			ACO data set (n=100)		COVID-19 data set (n=527)
**Sex, n (%)**					
	Male	37 (37)		301 (57.1)	
	Female	63 (63)		226 (42.9)	
Age (years), mean (SD)			78.8 (7.4)		52.6 (15)
**Minorities, n (%)**					
	Black	4 (4)		163 (30.9)	
	Hispanic	2 (2)		138 (26.2)	
	Asian	1 (1)		16 (3)	
	Indigenous	0 (0)		1 (0.2)	
College education, n (%)			51 (51)		160 (30.4)
Married, n (%)			50 (50.0)		195 (37)
**Clinical characteristics**					
	Number of encounters, median (min-max)	164 (8-858)		106 (1-2474)	
	PCP^b^ visit, n (%)	71 (71)		423 (80.3)	
	Dementia ICD^c^ code and medication, n (%)	51 (51)		166 (5.3)	

^a^NLP: natural language processing.

^b^PCP: primary care provider.

^c^ICD: International Classification of Diseases.

### Features of NAT

**Figure 1:**
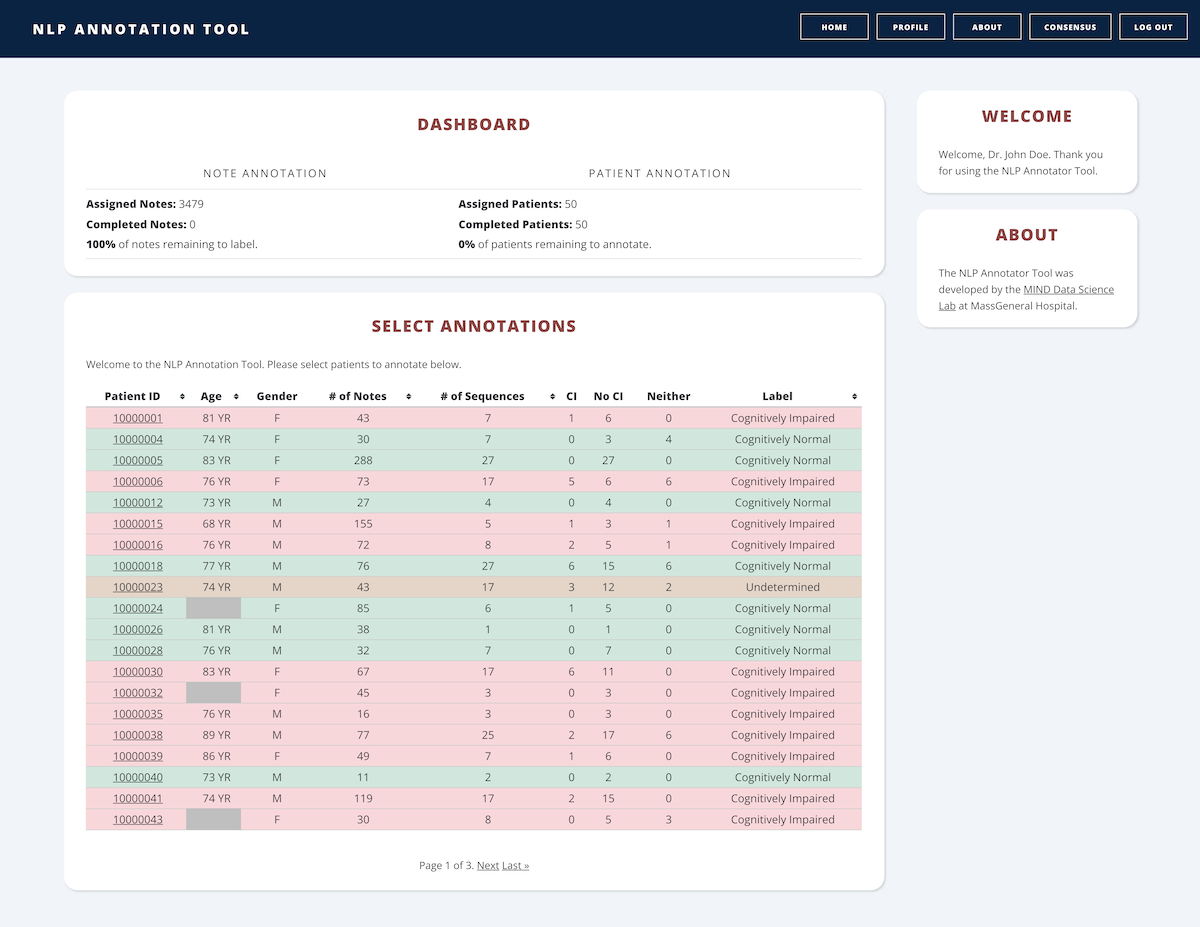
NAT dashboard: screenshot of the NAT dashboard displaying the current workload and assigned patients. A summary of patient information is displayed in each row, and the background reflects the cognitive status assigned to the patient. NAT: NLP annotation tool; NLP: natural language processing.

Upon logging in to our annotation tool, an authenticated user is presented with a dashboard listing the patient IDs, ages, and sexes of their assigned patients (Figure 1). In addition, the total number of notes, the sequences within the notes that match a cognition or ADL keyword (Multimedia Appendices 1 and 2), and the number of notes for each predicted class (ie, cognition and ADL) are also presented. After annotation, the patient’s label (CN, CI, or undetermined) is displayed with background colors reflecting the patient’s annotated cognitive status.

**Figure 2:**
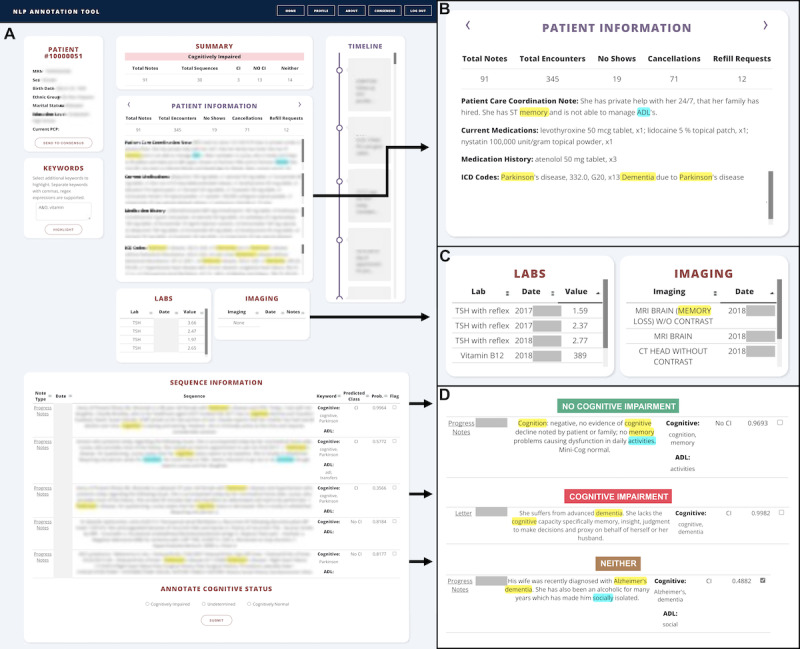
Annotation view: (A) patient view displaying summary information at the top and sequences from clinical notes at the bottom; (B) the Patient Information box summarizes health care interaction, patient care coordination notes, current medications, and diagnosis codes; (C) laboratory tests and imaging conducted on the patient; (D) sample sequences from notes with dementia and activities of daily living (ADLs) keywords highlighted. Each sequence is classified as cognitive impairment (CI), no CI, or neither, with a probability, and allows annotators to flag incorrect classifications.

Selecting a patient navigates the user to an annotation view summarizing the patient’s demographic and clinical information (Figure 2A). Engineered features, including the total number of notes, encounters, no shows, cancellations, and refill requests, along with the patient care coordination note (if any), diagnosis ICD codes, and medications, are displayed (Figure 2B). Brain imaging and relevant laboratory tests, such as thyroid-stimulating hormone or vitamin B12, allow annotators to consider systemic causes of cognitive changes (Figure 2C). Finally, notes sorted by the predicted probability and with highlighted keywords are presented to expedite the review of the entire chart history during the relevant period for the clinical adjudication of cognitive status. Examples of the three predicted classes of notes (CN, CI, and undetermined) are shown in Figure 2D.

### Evaluation of NAT

**Figure 3:**
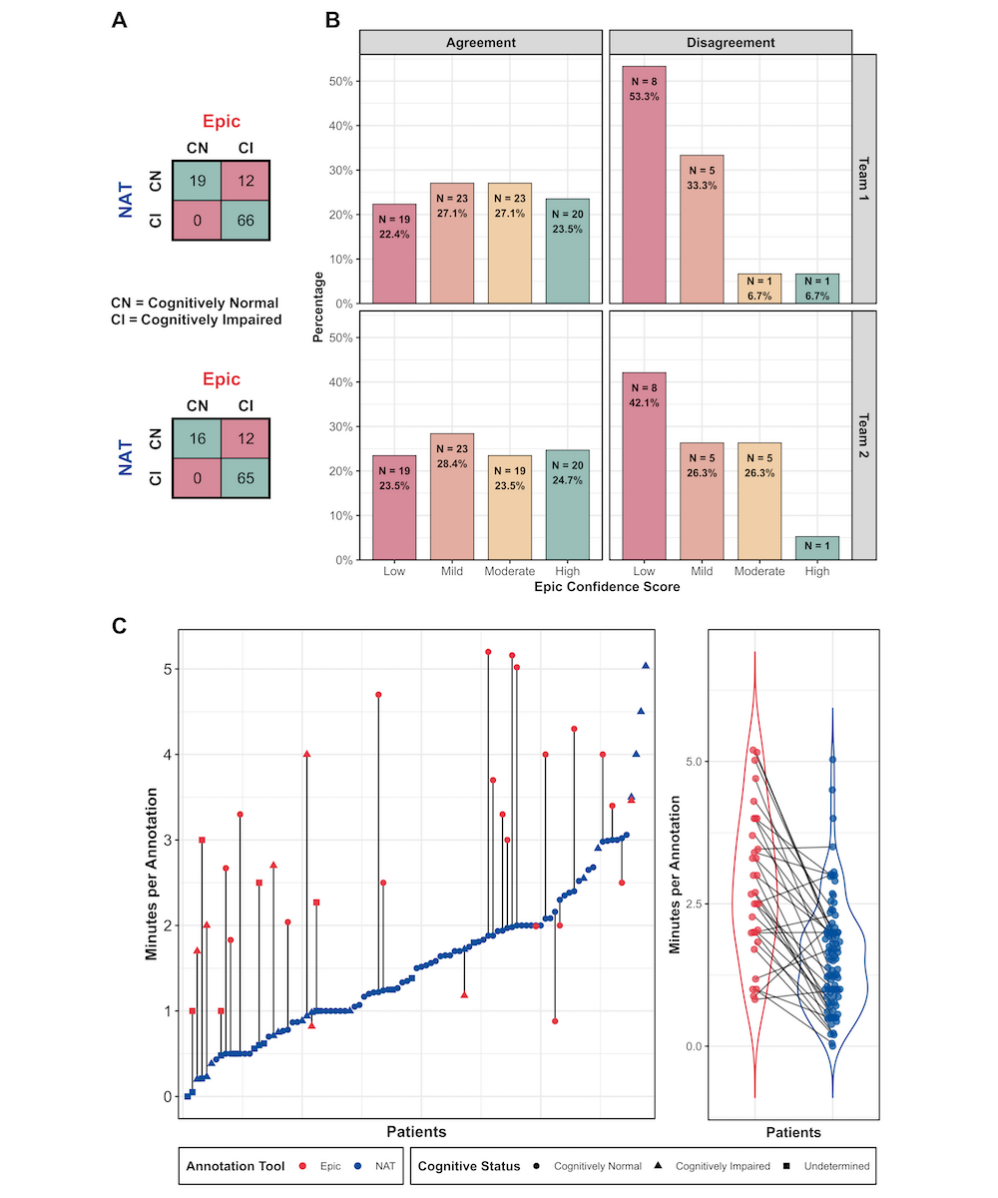
Comparison of adjudication with natural language processing (NLP)–powered annotation tool (NAT) and manual Epic chart reviews: (A) contingency table displaying adjudication with NAT versus Epic by team 1 (top row) and team 2 (bottom row); (B) distribution of confidence scores assigned in Epic manual chart reviews (Moura et al [15]) for agreements and disagreements between the two methods; (C) annotation time comparisons between NAT versus Epic.

Two teams of expert clinicians were randomly assigned patients and adjudicated the ACO data set, using NAT (team 1: LB, GKR, SSM; team 2: MBW and HA). We compared the phenotyping of cognitive status using NAT to manual chart reviews using Epic (labels were obtained from Moura et al [15]; patients who were not CN were grouped into the CI class). We removed patients annotated as “undetermined” in the set adjudicated using NAT, as they had little information in EHR to assess cognitive status and could not be directly compared to the labels obtained from Moura et al [15]. The agreement between NAT and manual Epic chart reviews was moderate for both team 1 (Cohen κ=0.68) and team 2 (Cohen κ=0.65) with a mean Cohen κ=0.67; the breakdown is shown in Figure 3A. Surprisingly, patients whose NAT label disagreed with the manual Epic chart reviews were annotated as CI using Epic and as CN using NAT. We manually reviewed the patients where the diagnostic labels disagreed; we found that NAT was able to highlight certain passages of text, such as “language, attention, and memory function are intact with good fund of knowledge”; the highlighted text facilitated the labeling of the patient as CN, whereas such phrases were easily missed in manual chart reviews. Moreover, if a patient had a transient cognitive deficit and was later evaluated as CN, for example, NAT presented all notes with highlighted evidence along with their dates in one view, making it easier to follow the sequence of events. The disagreements were mostly among patients annotated with a low confidence score in the Epic manually annotated data set [15] (Figure 3B). The interrater agreement of NAT adjudication between team 1 and team 2 was higher (Cohen κ=0.89) than the interrater agreement (Cohen κ=0.80) with manual Epic chart reviews reported in Moura et al [15].

**Figure 4:**
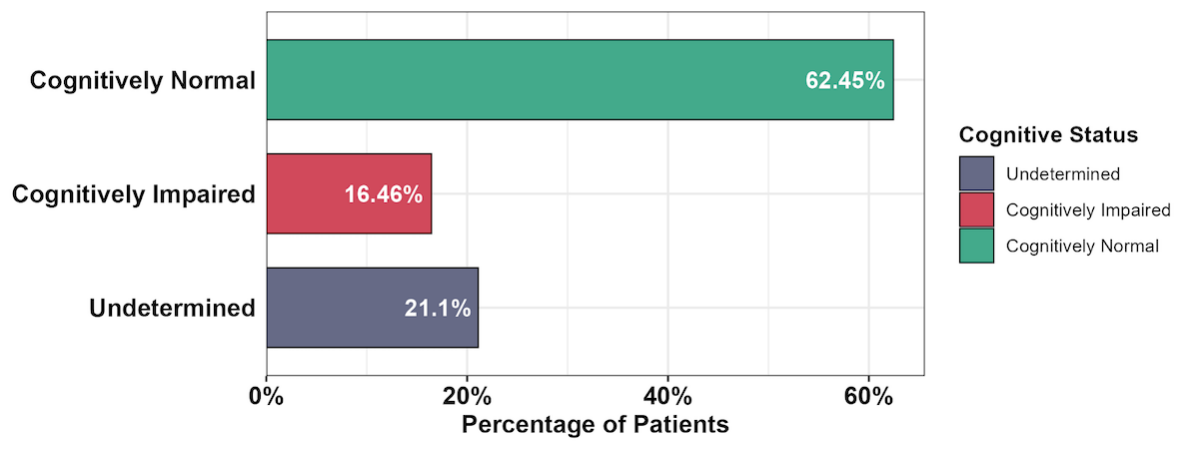
COVID-19 data set cognitive scores and distribution of cognitive scores in the COVID-19 data set.

Next, we compared the time required for phenotyping of cognitive status via NAT adjudication versus manual chart reviews in Epic. Four of the authors (DW, ER, HA, and SSM) adjudicated the full COVID-19 data set using NAT and recorded the annotation time for 129 patients. Two of the authors (HA and SSM) timed manual chart reviews in Epic for 32 randomly sampled patients. To ensure that a patient was not adjudicated using both methods by the same person, HA used Epic to perform chart reviews of patients adjudicated by SSM using NAT and vice versa. For most of the patients, the annotation time was substantially shorter with NAT as compared to manual chart reviews in Epic (Figure 3C). Adjudications using NAT provided substantial speed-up of annotations compared to manual chart reviews in Epic (time difference mean 1.4, SD 1.3 minutes; *P*<.001; ratio median 2.2, min-max 0.4-20). Additionally, we observed that clinicians spent more time using NAT on the first half of patients compared to the second half. This “learning effect” was not observed with manual Epic chart reviews. The breakdown of the cognitive status for the COVID-19 data set is shown in Figure 4. Notably, the cognitive status for 21.1% (n=111) of patients was undetermined, suggesting that there was little information in EHR to determine their cognitive status.

## Discussion

### Principal Findings

In this study, we developed and evaluated a novel semiautomated NLP-powered annotation tool, NAT, to facilitate phenotyping of cognitive status. Clinical experts adjudicated the cognitive status of 627 patients at MGB health care using NAT or traditional chart reviews. NAT improves the efficiency and interrater reliability of chart review as compared to manual adjudication.

### Strengths

Phenotyping methods have been applied to EHR to successfully identify patients with autism [28], diabetes [29], immunological diseases [30], and several chronic diseases [16]. EHR has been extensively used for dementia research, but the outcomes are typically defined by diagnosis codes or specialist diagnoses. Although phenotyping tools using NLP have been developed to detect cognitive impairment [18,19,24], they have been limited by their performance. In this study, we propose a novel semiautomated approach that combines NLP outputs with manual adjudication.

We selected this approach as it combines the automation of an NLP tool and the expert review required for phenotyping cognitive status. Phenotyping cognitive status requires the input from both structured (eg, diagnosis codes and medications) and unstructured (eg, clinical notes and images) data, and currently, there are no machine learning tools that integrate multiple data modalities. The approach has several advantages over manual chart reviews. Cognitive concerns are often subjective, and a significant amount of information is required to confidently ascertain the correct diagnosis. Since diagnoses are staged across months or years, individual notes across time must be evaluated together—NAT filters data for the period of interest and thus facilitates the adjudication process. Next, the absence of cognitive deficits is often difficult to adjudicate with confidence. In these cases, the annotator needs to review all notes to ensure there were no signs of cognitive impairment. NAT improves the efficiency of such tasks, as it automatically flags notes with signs of cognitive impairment as well as those with information on normal cognition and ranks them in order of importance. In addition, clinicians often use a wide variety of terms and phrases in clinical notes that can easily be missed in manual reviews. NAT, on the other hand, highlights all cognition-related patterns and phrases, decreasing the likelihood that the annotator might miss any information relevant to the decision-making task. Finally, NAT streamlines an established adjudication protocol and thus improves interrater agreement. NAT can, in principle, be extended to local hospitals and clinics that have digitized data but not an EHR system.

### Limitations

This study has several limitations. First, NAT does not link to brain images, which may contain information relevant to brain function. Second, although NAT improves the efficiency of adjudicating cognitive status compared to manual chart reviews, it is not scalable to large data sets of thousands of patients. To scale to such sample sizes, fully automated machine learning algorithms that replicate the adjudication process are required. In the future, we plan to use NAT to create gold-standard data sets for training and validation of such machine learning algorithms for phenotyping cognitive status. Third, NAT adjudication was evaluated on data from a single health care system. Whether the cognition and ADL-related keywords apply to other health care settings is yet to be confirmed. The performance of the NLP tool [26] also needs to be evaluated with external data. Fourth, adjudicators were not blinded to identifiable information in EHR, which may have introduced biases in their labels. Tools, such as Philter, could be used in the future to remove protected health information in NAT [31]. Finally, research studies using EHR-based data sets are limited by the information available within the health care system and miss records of care outside the system. Such patients with missing information were labeled as “undetermined” in this study, but studies that use diagnosis codes for phenotyping of cognitive status may incorrectly label such patients as CN instead of distinguishing them as patients with insufficient information. Our study highlights the issue of missing information when phenotyping cognitive status in EHR, and consequently, the need for future work to minimize biases if such patients are excluded in a research study.

### Conclusions

Although there is no substitute for a longitudinal cohort with formal cognitive evaluations to study Alzheimer disease and related dementias, leveraging EHR data with NLP holds promise. In this diagnostic study, we developed and evaluated a semiautomated NLP-powered annotation tool, NAT, to facilitate the phenotyping of cognitive status in EHRs. Expert clinicians adjudicated cognitive status of 627 patients from two distinct data sets; NAT had a high interrater agreement and improved the speed of annotations compared to manual chart reviews. Using NAT to adjudicate cognitive status would likely increase the feasibility and scalability of building gold-standard data sets for machine learning algorithms and research cohorts to study cognitive decline.

## Appendix

Multimedia Appendix 1 Data query, preparation, and preprocessing steps.

Multimedia Appendix 2 Regular expressions of dementia-related keywords.

Multimedia Appendix 3 Regular expressions of activities of daily living (ADLs) keywords.

Multimedia Appendix 4 Data model.

## Abbreviations

ACO: Accountable Care Organization
ADL: activities of daily living
CI: cognitively impaired
CN: cognitively normal
EHR: electronic health record
ICD: International Classification of Diseases
MCI: mild cognitive impairment
MGB: Mass General Brigham
NAT: NLP annotation tool
NLP: natural language processing
