# Supplementary figures and images for: Development and Evaluation of a Natural Language Processing Annotation Tool to Facilitate Phenotyping of Cognitive Status in Electronic Health Records: Diagnostic Study
Source: J Med Internet Res. 2022 Aug 30;24(8):e40384. doi: 10.2196/40384 (PMC9472045; doi:10.2196/40384)

## Preparation

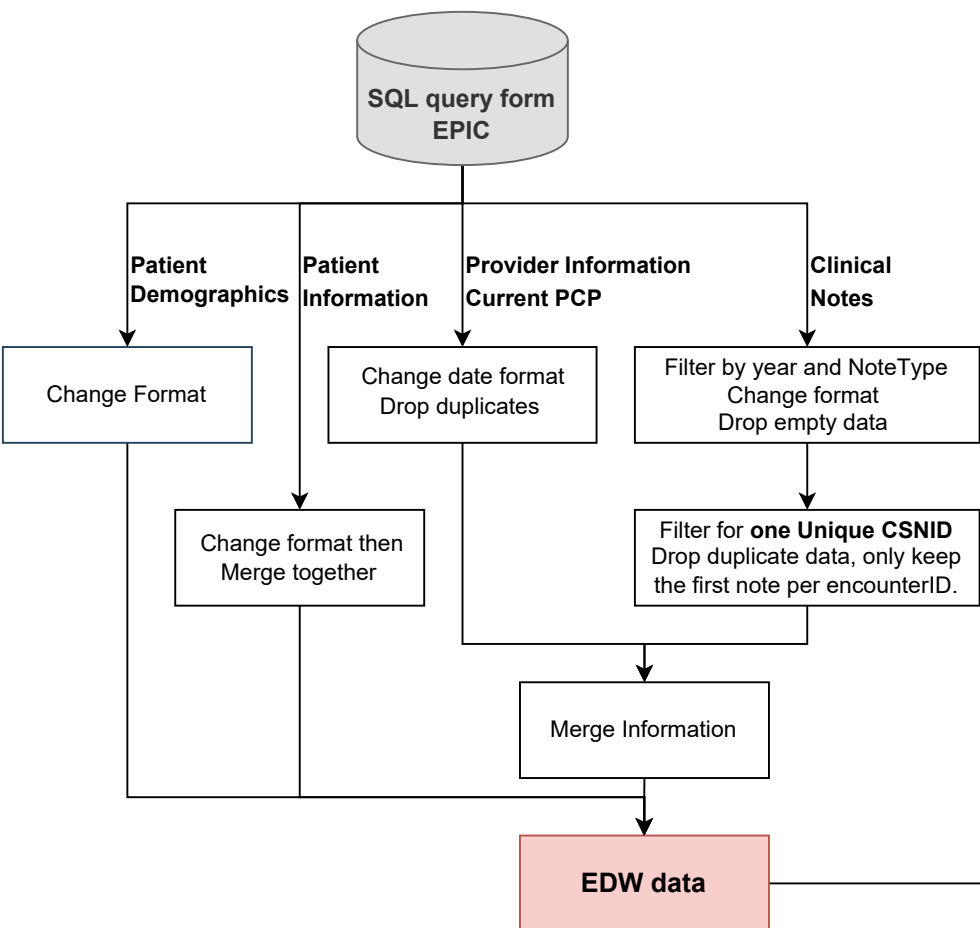

## Pre-Processing Pipeline

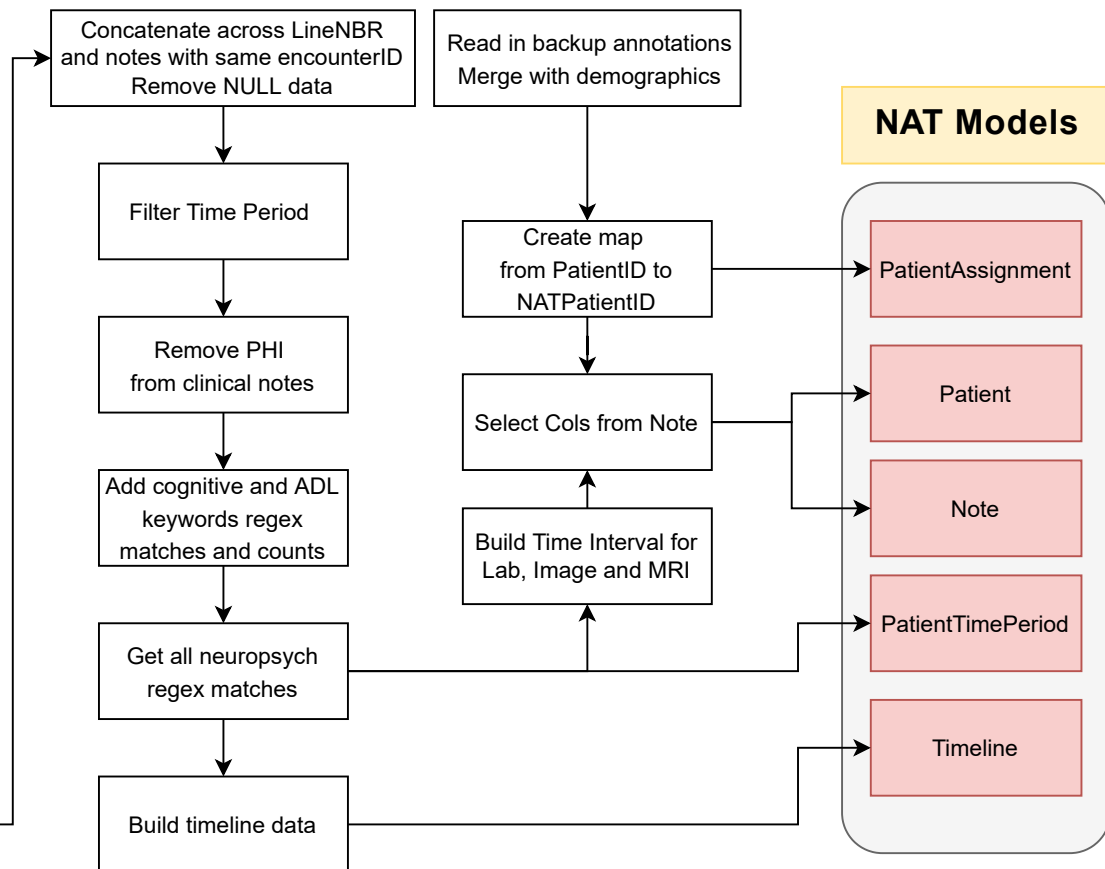

Supplement: Multimedia Appendix 1 [file jmir_v24i8e40384_app1.pdf]

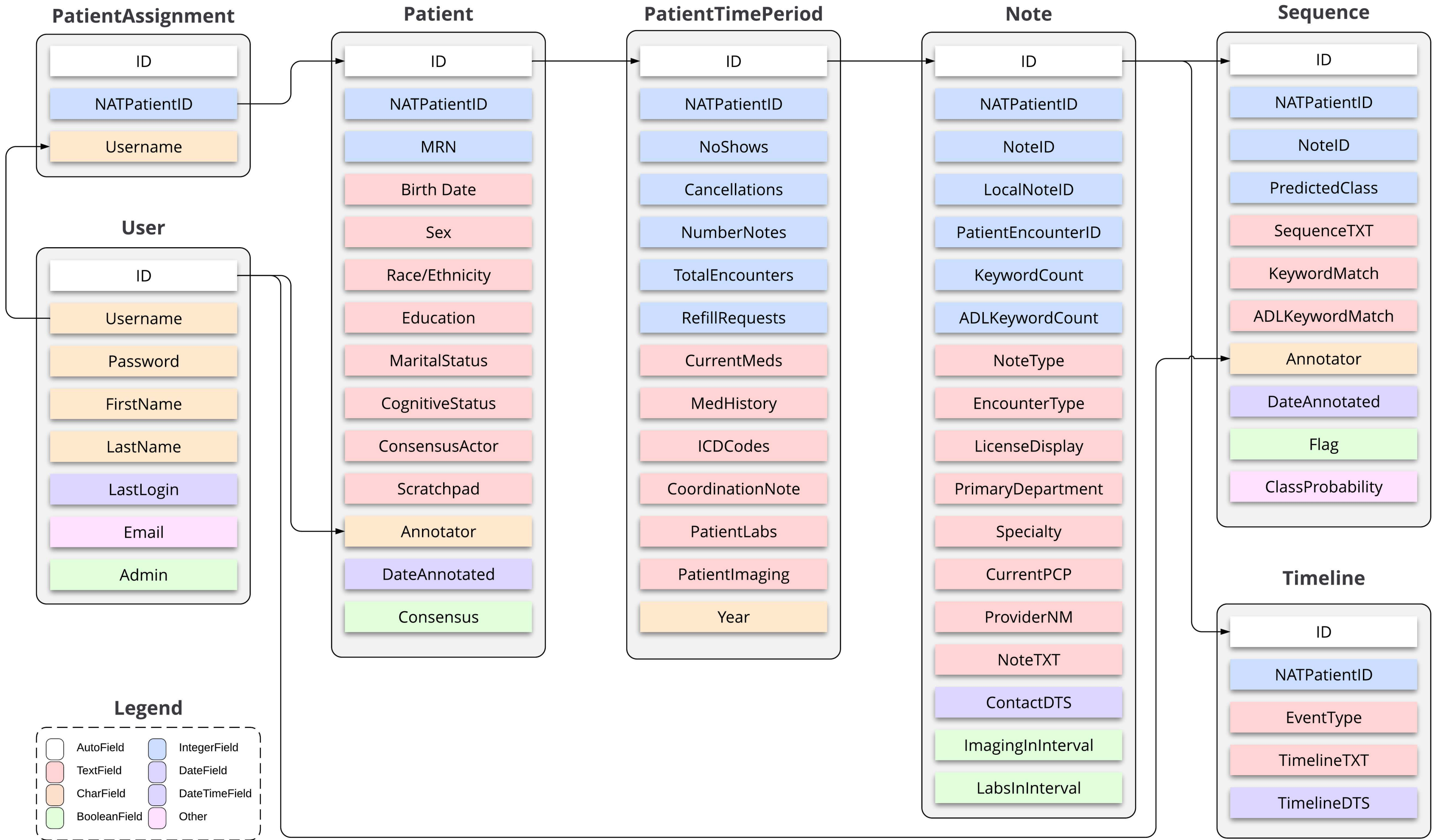

Supplement: Multimedia Appendix 4 [file jmir_v24i8e40384_app4.pdf]
